# Complete Genome Sequence of Bovine Papillomavirus Genotype 13 from Local Yellow Cattle in Hainan Province, China

**DOI:** 10.1128/genomeA.01087-14

**Published:** 2014-12-04

**Authors:** Feng Pang, Qiaoyun Shi, Li Du, Tianjing Zhao, Ying Cheng, Hanwei Jiao, Jianguo Zhao, Manchuriga Wang, Hui Rong, Hailong Zhou, Fengyang Wang

**Affiliations:** College of Agriculture, Hainan University, Hainan Key Laboratory of Tropical Animal Reproduction & Breeding and Epidemic Disease Research, Animal Genetic Engineering Key Laboratory of Haikou, Haidian Island, Haikou, China

## Abstract

Here, we present the complete genome sequence of bovine papillomavirus genotype 13 isolated from local yellow cattle in Hainan, China. The genome is 7,961 bp and contains six early genes and two late genes. This analysis provides important information for the research of bovine papillomavirus (BPV) in China.

## GENOME ANNOUNCEMENT

As small, nonenveloped, double-stranded DNA (dsDNA viruses), papillomaviruses (PVs) are involved in the pathogenesis of human and animal tumors, including those in cattle (1). According to genome nucleotide sequence similarities, 13 bovine papillomavirus (BPV) types (BPV-1 to -13) have been distinguished, and these have been assigned to four genera, *Deltapapillomavirus*, *Epsilonpapillomavirus*, *Xipapillomavirus*, and an unassigned genus. BPV-1, -2, and-13 belong to the genus *Deltapapillomavirus*, and BPV-1 and BPV-2 infect the epithelium and dermis, giving rise to fibropapillomas (2, 3). BPV-5 and -8 belong to the genus *Epsilonpapillomavirus*, and BPV-5 infects the epithelium and dermis, inducing fibropapillomas and true epithelial papillomas of the skin. BPV-3, -4, -6, -9, -10, -11, and-12 belong to the genus *Xipapillomavirus*. BPV-3, BPV-4, and BPV-6 are strictly epitheliotropic, inducing epithelial papillomas. BPV-7 belongs to an unassigned genus (4–8).

The complete genome of BPV consists of the early region, the late region, and the noncoding region (NCR) between them. The early region of the BPV genome encodes viral regulatory proteins, which are necessary for the initiation of virus replication. The late region contains the major (L1) and minor (L2) capsid protein genes (8, 9).

To identify the type of the bovine papillomavirus, the primers L1-F and L1-R were used to amplify the L1 fragment, which is used for BLAST. Phylogenetic analysis with the L1 fragment showed that BPV Hainan strain belongs to BPV- 13.

According to the genome information of BPV genotype 13 (GenBank accession no. JQ798171), seven pairs of primers were designed to amplify the complete genome sequence of the BPV Hainan strain. Based on the sequencing results, the complete genome sequence of the BPV Hainan strain was obtained. The genomic nucleotide sequence was deposited in GenBank using the Sequin software from the National Center for Biotechnology Information (NCBI), under accession no. KM258443.2. Putative open reading frames (ORFs) were identiﬁed using the ORF Finder tool (http://www.ncbi.nlm.nih.gov/gorf/gorf.html) and compared with the BPV-1, -2, -3, -4, -6, -9, -10, -11, -12, and -13 genomes.

The size of the BPV type 13 Hainan strain is 7,961 bp, containing E1, E2, E4, E5, E6, E7, L1, L2, and NCR. In comparison with the genomes of BPV-1 (GenBank accession no. X02346), BPV-2 (M20219), BPV-3 (AF486184), BPV-4 (X05817), BPV-5 (AF457465), BPV-6 (AJ620208), BPV-7 (DQ217793), BPV-8 (DQ098913), BPV-9 (AB331650), BPV-10 (AB331651), BPV-11 (AB543507), BPV-12 (JF834523), and BPV-13 (JQ798171), the nucleotide sequence of BPV Hainan strain has 87.27%, 90.83%, 41.57%, 43.26%, 48.44%, 43.04%, 43.26%, 47.62%, 45.77%, 38.79%, 43.25%, 40.86%, and 99.75% homology, respectively.

The cloning and sequencing of the complete genome of BPV genotype 13 Hainan strain from local yellow cattle will provide valuable information for the research of BPV in China.

### Nucleotide sequence accession number.

The completed genome sequence of the BPV genotype 13 Hainan strain has been deposited in GenBank under accession no. KM258443.
